# Emphysematous cystitis: A case report and literature review

**DOI:** 10.1002/ccr3.2980

**Published:** 2020-05-28

**Authors:** Alain Mwamba Mukendi

**Affiliations:** ^1^ Department of Urology Chris Hani Baragwanath Academic Hospital University of the Witwatersrand Johannesburg South Africa

**Keywords:** emphysematous cystitis, gas forming bacteria, infectious complication, urinary tract infection

## Abstract

Emphysematous cystitis is a rare potentially life‐threatening condition and a distinct type of complicated lower urinary tract infection generally associated with diabetes mellitus and diagnosed radiologically. This case report presents a case of emphysematous cystitis as post hiatal hernia repair infectious complication incidentally found on CT abdomen/pelvis.

## Graphical abstract

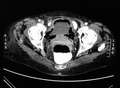

## 1 INTRODUCTION

Emphysematous cystitis is a less frequent clinical entity most commonly associated with Diabetes mellitus which is considered to be the major risk factor. Other risk factors include urinary tract outlet obstruction, neurogenic bladder, immune‐deficiency, indwelling urethral catheters, and chronic UTIs.^1^ The pathogenesis is still poorly understood and the most associated causative pathogens are gas forming bacteria such as *Escherichia coli, Klebsiella pneumoniae, and Enterococci*.^1^, ^2^ Affected patients may be symptomatic or not with the degree of inflammation not necessarily correlating with the clinical presentation.^2^ Our case finds its uniqueness in highlighting the occurrence of emphysematous cystitis without any of the aforementioned common risk factors as post hiatal hernia repair infectious complication.

## 2 CASE REPORT

**Figure 1:**
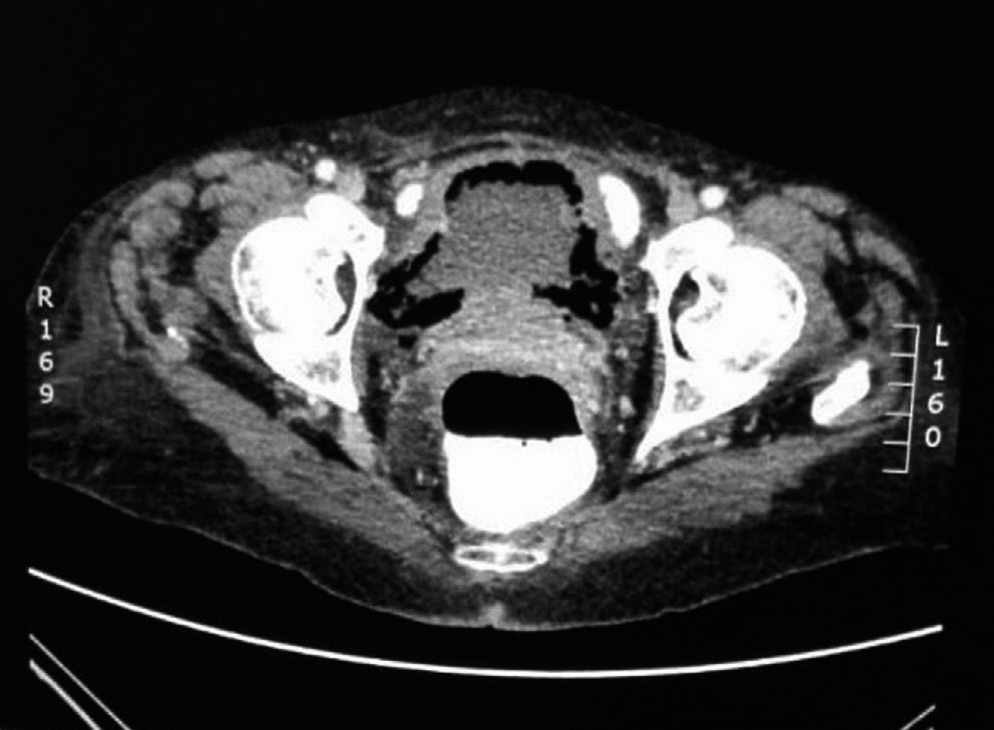
Axial view of CT abdomen/pelvis demonstrating the presence of extensive air in the bladder wall

**Figure 2:**
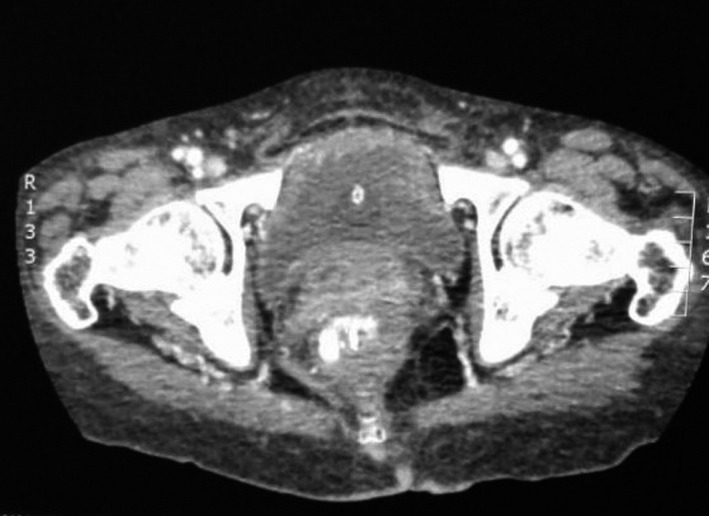
CT scan images showing resolution of emphysematous cystitis after 10 days of antibiotics

A 65‐year‐old female hypertensive, nondiabetic admitted in the surgical ward with gastroatmospheric fistula and sepsis complicating an open hiatal hernia repair. She underwent two previous failed laparoscopic hiatal hernia repairs and was 4‐week postopen repair. She had no history of smoking or alcohol consumption, no known allergies, no history of neurogenic bladder, urinary tract outlet obstruction, or chronic urinary tract infection. She was married and never held any employment. She denied any suprapubic pains or any lower urinary tract symptoms. On physical examination, she was found to be febrile with a temperature of 38.4°C, her pulse was 112 beats/min and blood pressure 99/61. Examination also revealed a laparotomy scar with an epigastric dehiscence exposing a part of the stomach which has a small defect. Her C ‐ reactive protein (CRP) was 249 mg/L (normal: <10 mg/L) and white blood cell (WBC) count 25 × 10^9^/L (normal: 3.9‐10 × 10^9^/L) She was empirically started on Piperacillin/tazobactam 4.5 g 6 hourly. CT Abdomen/pelvis done to look for abdominal collection as source of sepsis found no intra‐abdominal collection but a gastroatmospheric fistula and extensive emphysematous cystitis (Figure 1) reason for urological consult. Antibiotics were changed to Ceftriaxone 1 g IVI 12 hourly due to poor response to Piperacillin/tazobactam and a Foley's catheter inserted. Urinalysis was unfortunately done after antibiotic therapy has already been started and showed only leukocytosis; urine microscopy, culture and sensitivity were also done and showed pyuria, no growth after 1 day and confirmed the presence of antimicrobial substances. Septic markers improved (CRP 67 mg/L and WBC 10.99 × 10^9^/L) on ceftriaxone and bladder drainage with a catheter. Noncontrast CT scan done after 10 days of antibiotics demonstrated resolution of the emphysematous cystitis (Figure 2). A repeat urine microscopy, culture, and sensitivity at 2‐week postantibiotic therapy showed no leukocytes and no bacterial growth. At 6‐month follow‐up, bedside ultrasound showed normal bladder thickness and urinalysis was completely normal.

## 3 DISCUSSION/CONCLUSION

Emphysematous cystitis is a severe urinary tract infection characterized by build‐up of gas within the bladder wall or lumen as a result of fungal or bacterial fermentation.^3^ *Escherichia coli* was found to be the most prevalent microorganism followed by *Klebsiella pneumonia*. Other gas‐producing microorganisms include *Enterococcus, Candida, and Clostridium perfringens*.^2^ The patient reported here had a confirmed emphysematous cystitis on CT scan. She was unfortunately already on antibiotics when urine culture and sensitivity was sent as demonstrated on urine results with a negative culture.

The majority of affected patients are female, elderly, and diabetic with clinical presentation ranging from asymptomatic to symptomatic and severe forms such as peritonitis or septic shock.^2^, ^3^ When symptomatic, patients may report abdominal pain; frequency of urination; dysuria, pneumaturia, and rarely subcutaneous emphysema.^4^, ^5^, ^6^ Our patient was a female nondiabetic who did not experience any lower urinary tract symptoms. However, nondiabetic patients can also be affected through a different pathogenesis pathway as described below.

The pathogenesis of emphysematous cystitis is still not yet fully understood hence there are numerous theories. Nevertheless, the production of gas within the affected tissues seems to be mostly associated with a multifactorial etiology of impaired host responses with sugar or protein fermentation. The presence in a tissue of gas‐producing organisms combined with a high glucose concentration and impaired tissue perfusion support the development of emphysematous infections. In addition, the high glucose concentration within the tissue acts as a substrate for pathogens to produce carbon dioxide (CO_2_) through natural fermentation processes in diabetic patients. However, in nondiabetic patients urinary albumin acts as the favorable substrate for gas production by microorganisms within the tissue. An impaired host response, involving impaired catabolism and vascular compromise within the tissues, constitutes another suggested theory for gas production.^6^ We believe that our patient may probably have developed this condition because of postsurgical impaired host response as she was nondiabetic and urinalysis did not show any proteins.

Radiographic Imaging remains the only diagnostic tool which can demonstrate a rim of gas lucency outlining the bladder wall and/or air fluid levels within the bladder on plain abdominal X‐rays. However, computed tomography (CT) is more accurate in detecting non apparent cases on plain radiography, in defining the extent and severity of the disease, in differentiating emphysematous cystitis from other conditions such as colovesical fistula, intra‐abdominal abscesses, and assessing possible development of ascending infections such as emphysematous pyelonephritis.^3^, ^4^

It is, however, important to be reminded that it is a potential life‐threatening disease because of rapid progression to bladder necrosis, emphysematous pyelonephritis, urosepsis, and death. Therefore, prompt evaluation and early treatment are necessary.^2^

Emphysematous cystitis is managed with aggressive broad spectrum parenteral antibiotics until the sensitivities of the isolated microorganisms are known and switched to more specific ones; bladder drainage with a catheter; tight glycemic control and treatment of any underlying comorbid disorders.^2^, ^3^, ^4^ If there is no response to conservative treatment, surgical management is needed with options ranging from debridement, partial cystectomy, cystectomy.^2^ Our patient responded well to medical treatment as demonstrated by improved septic markers and computed tomography resolution of features suggestive of emphysematous cystitis and therefore did not require any surgical intervention.

In conclusion, emphysematous cystitis is a rare entity known to affect primarily patients with uncontrolled diabetes mellitus. However, nondiabetic patients can also be affected even though uncommon. This report appears to be the first of its kind associating emphysematous cystitis to post hiatal hernia repair complications. The prevalence of this condition could be underestimated as radiological assessment is required for its diagnosis which is not routinely done in all patients with urinary tract infection. We believe that Clinicians and radiologists should be aware or reminded of the importance of radiological evaluation of patients with urinary tract infection as an adjunct tool to detect emphysematous cystitis especially when there is high index of suspicion.

## CONFLICT OF INTEREST

None declared.

## AUTHOR CONTRIBUTIONS

AMM: Substantial contributions to conception and design of the case report, acquisition of data, drafting of the manuscript, critical revision for important intellectual content, and approval of final version.

## CONSENT

Written informed consent was obtained from the patient for publication of this manuscript and accompanying pictures. A copy of the written consent is available for review by the Editor‐in‐Chief of this journal.
